# Effects of Different Drug Combinations in Immunodeficient Mice Infected with an Influenza A/H3N2 Virus

**DOI:** 10.3390/microorganisms8121968

**Published:** 2020-12-11

**Authors:** Zeineb Mhamdi, Hugues Fausther-Bovendo, Olus Uyar, Julie Carbonneau, Marie-Christine Venable, Yacine Abed, Gary Kobinger, Guy Boivin, Mariana Baz

**Affiliations:** Research Center in Infectious Diseases of the CHU of Québec, Laval University, Québec City, QC G1V 4G2, Canada; zeineb.mhamdi.1@ulaval.ca (Z.M.); hugues.fausther.bovendo@crchudequebec.ulaval.ca (H.F.-B.); Olus.Uyar@crchudequebec.ulaval.ca (O.U.); julie.carbonneau@crchudequebec.ulaval.ca (J.C.); marie-christine.venable@crchudequebec.ulaval.ca (M.-C.V.); yacine.abed@crchudequebec.ulaval.ca (Y.A.); gary.kobinger@crchudequebec.ulaval.ca (G.K.); guy.boivin@crchudequebec.ulaval.ca (G.B.)

**Keywords:** influenza, H3N2, immunosuppression, combination therapy, oseltamivir, favipiravir, baloxavir marboxil, resistance, mice

## Abstract

The prolonged treatment of immunosuppressed (IS) individuals with anti-influenza monotherapies may lead to the emergence of drug-resistant variants. Herein, we evaluated oseltamivir and polymerase inhibitors combinations against influenza A/H3N2 infections in an IS mouse model. Mice were IS with cyclophosphamide and infected with 3 × 10^3^ PFU of a mouse-adapted A/Switzerland/9715293/2013 (H3N2) virus. Forty-eight hours post-infection, the animals started oseltamivir, favipiravir or baloxavir marboxil (BXM) as single or combined therapies for 10 days. Weight losses, survival rates and lung viral titers (LVTs) were determined. The neuraminidase (NA) and polymerase genes from lung viral samples were sequenced. All untreated animals died. Oseltamivir and favipiravir monotherapies only delayed mortality (the mean day to death (MDD) of 21.4 and 24 compared to 11.4 days for those untreated) while a synergistic improvement in survival (80%) and LVT reduction was observed in the oseltamivir/favipiravir group compared to the oseltamivir group. BXM alone or in double/triple combination provided a complete protection and significantly reduced LVTs. Oseltamivir and BXM monotherapies induced the E119V (NA) and I38T (PA) substitutions, respectively, while no resistance mutation was detected with combinations. We found that the multiple dose regimen of BXM alone provided superior benefits compared to oseltamivir and favipiravir monotherapies. Moreover, we suggest the potential for drug combinations to reduce the incidence of resistance.

## 1. Introduction

Influenza viruses (IVs) are among the most important pathogens causing severe respiratory infections. During seasonal influenza epidemics, the A/H3N2 subtype can be associated with higher mortality and morbidity compared to other influenza subtypes [1]. Two classes of antivirals targeting the viral neuraminidase (NA) and the polymerase complex are currently clinically available for the treatment of influenza infections. Oseltamivir phosphate (OS), a NA inhibitor (NAI), is the most frequently prescribed compound and has demonstrated efficacy against influenza A and B viruses [2,3].

In 2018, the novel antiviral, baloxavir marboxil (BXM) was approved in the USA, Japan and several other countries for the treatment of uncomplicated influenza in otherwise healthy individuals [4,5]. Its active form, baloxavir acid (BXA), potently inhibits the influenza cap-dependent endonuclease encoded by the PA gene, preventing viral replication [6].

Treatment with BXM significantly improved the time of influenza symptom alleviation compared with placebo as well as reduced the viral load after a single dose [7]. Furthermore, it decreased the duration of virus shedding more rapidly than OS in otherwise healthy (CAPSTONE-1) and high-risk (CAPSTONE-2) patients [7,8], including adults older than 65 years or those who have conditions such as asthma, chronic lung disease, morbid obesity, or heart disease. Another polymerase inhibitor, favipiravir (FA; T-705), was approved in 2014 in Japan, but its use is restricted to patients with novel or re-emerging pandemic IVs and in cases of NAI resistance [5,9]. FA is a nucleoside analog that targets the polymerase basic 1 (PB1) protein, resulting in errors during viral RNA synthesis [10]. This drug displays a high genetic barrier to resistance, with one exceptional case of in vitro resistance mediated by a K229R substitution in the PB1 protein of A/H1N1 virus [11].

Virus clearance is usually delayed among high-risk populations, including immunosuppressed (IS) patients [12,13]. To inhibit virus replication in these individuals, the prolonged use of NAIs is required, which frequently leads to the emergence of drug-resistant viruses [13,14,15,16,17,18,19]. The substitutions R292K and E119V in the NA of A/H3N2 viruses have been reported in clinical isolates with reduced susceptibility to OS [13,20,21]. In clinical trials, mutant viruses harboring the I38T/M/F PA substitutions with reduced susceptibility to BXM were reported [7]. The emergence of these variants could be problematic, especially in IS patients with prolonged viral shedding.

Interestingly, a few studies have found beneficial effects of combination therapy compared with monotherapy in inhibiting IVs in IS hosts [22,23]. The evaluation of combination treatment may be done using appropriate animal models, including those that mimic viral replication in IS patients. However, very few studies have examined the potential benefits of the combination therapy in IS animal models [24,25].

In this study, we used a mouse model of immunosuppression by serial administrations of cyclophosphamide (CP), a drug commonly used in antitumor therapy [26,27,28]. First, we confirmed that the lymphoproliferative response and number of total T, B and neutrophil cells was reduced. Then, we used such an IS mouse model and we evaluated whether the combination of OS and polymerase inhibitors (FA or BXM) would improve therapeutic efficacy and reduce the emergence of resistance mutations compared with single therapy following lethal infection with a contemporary mouse-adapted influenza A/H3N2 virus [29].

In the present study, we showed the superior efficacy of BXM single therapy with repeated doses compared to OS and FA monotherapies during A/H3N2 infections in IS mice. We found a synergistic interaction between OS and FA combination therapy over monotherapies. In addition, our data suggest the potential for drug combinations to reduce the incidence of resistance among IS patients.

## 2. Materials and Methods

### 2.1. Cells and Virus

Madin–Darby canine kidney cells overexpressing the α2,6 sialic acid receptor (ST6-GalI-MDCK cells) were kindly provided by Y. Kawaoka from the University of Wisconsin, Madison, WI [30]. The mouse-adapted A/Switzerland/9715293/2013 (H3N2) IV was generated in our laboratory [29]. Virus stocks were prepared, titrated in ST6-GalI-MDCK cells and stored at −80 °C.

### 2.2. CP Treatment of Mice and Confirmation of Immunosuppression

To confirm CP-induced immunosuppression, female C57BL/6 mice (Charles River, St-Constant, Quebec, Canada) were infected intranasally (i.n.) under isoflurane anesthesia with 3 × 10^3^ PFU of the A/Switzerland/9715293/2013 (H3N2) virus in 50 μL of PBS. A group of mice received 50 μL of PBS and served as control. Mice received either saline or CP (Sigma, St-Louis, MO) (100 mg/kg) intraperitoneally (i.p.), 24 h before infection, and on days 3, or 7 post-infection (p.i.), as previously described [24]. Groups of 3 mice were euthanized on days 4 and 10 p.i. and spleens were removed to quantify immune cells and proliferation capacity. For proliferation assays, splenocytes were stained with 5 μM carboxyfluorescein succinimidyl ester (CFSE) according to the manufacturer’s instructions (Thermo Fisher Scientific, Burlington, Canada). and stimulated with antibodies against CD3ε (1 μg/mL) and CD28 (1 μg/mL) monoclonal antibodies (BD Biosciences, Sanjose, CA) for T cells stimulation, resiquimod (R848) (1 μg/mL) to stimulate B cells and IL-15 (100 ng/mL) to induce natural killer (NK) cells proliferation (Sigma Aldrich, Ontario, Canada). After 5 days, the cells were stained with anti-CD3ε, B220 or NK1.1 antibodies (BD Biosciences, Sanjose, CA). Cell proliferation was analyzed by flow cytometry (LSR-II, BD Biosciences). To quantify the immune cells, splenocytes were incubated with a cocktail of antibodies (Table S1) for 30 min to identify the different immune subsets. Flow cytometry data acquisition and analyses were performed using a BD SORP LSR II (BD Biosciences) software.

### 2.3. Antiviral Compounds

Oseltamivir-phosphate (Tamiflu) was purchased from a local pharmacy and oseltamivir-carboxylate was synthesized by Hoffmann-La Roche (Basel, Switzerland). Both compounds were suspended in sterile water. Favipiravir was purchased from BOC Sciences (Shirley, NY, USA) and prepared in sterile water supplemented with 74.6 mg/mL of meglumine excipient. Baloxavir marboxil (HY-109025A) was purchased from MedChem Express (Princeton, NJ, USA) and prepared with 0.5% methylcellulose.

### 2.4. Influenza A/H3N2 Infections in IS Mice

Animal experiments were approved by the Animal Care Ethics Committee of Université Laval and the mice were used in accordance with the guidelines of the Canadian Council on Animal Care. Groups of seventeen 6–8 week-old female C57BL/6 mice housed four to five per cage and kept under conditions which prevented cage-to-cage infections, were treated i.p. with 100 mg/kg of CP 24 h before infection and on days 3, 7, 11, 15, 19, 23, and 27 p.i. On day 0, mice were infected i.n., under isoflurane anesthesia, with 3 × 10^3^ PFU of A/H3N2 virus in 50 µL of PBS. Treatments were started 48 h p.i. by oral gavage for 10 days with single drug regimen: OS (20 mg/kg; twice a day (BID)), FA (100 mg/kg; BID), and BXM (40 mg/kg; once a day); bi-therapy: OS and FA or OS and BXM or tri-therapy: OS, FA and BXM. A group of animals inoculated with the virus were treated with meglumine for 10 days. A group of 4 uninfected mice served as the control. Animals were weighed daily for 28 days and mice with weight losses ≥ 20% were humanely euthanized. Four mice per group were sacrificed on days 7 and 15 p.i. and lungs were removed aseptically. For the determination of lung viral titers (LVTs), harvested lung tissues were homogenized in 1 mL of PBS containing 2× antibiotic–antimycotic solution (penicillin, streptomycin and amphotericin B) using Omni Tip homogenizer (OMNI International, Kennesaw, GA, USA). Cells were pelleted by centrifugation (600× *g*, 5 min) and supernatants were used for the determination of TCID_50_ titers using ST6-GalI-MDCK cells [31].

### 2.5. Sequencing of Viral Genes

Viral NA, polymerase acidic (PA), and polymerase basic 1 (PB1) genes were amplified by reverse transcription (RT)-PCR from lung homogenates (collected at day 15 p.i.) using specific primers (available upon request) and sequenced using the ABI 3730 DNA Analyzer (Applied Biosystems, Carlsbad, CA, USA) [24].

### 2.6. Droplet Digital RT-PCR (RT-ddPCR)

RNA was isolated from lung homogenates as previously described [24]. The RT-ddPCR workflow and data analyses were performed to assess the presence of PA/I38T substitution with the One-Step RT-ddPCR Advanced Supermix (Bio-Rad Laboratories, Mississauga, ON, Canada) according to the manufacturer’s instructions and as previously described [32,33]. The primers and probes targeting the I38 wild-type (WT) and T38 (mutant) variants for the A/H3N2 virus are available upon request. The cycled plate was then transferred and read in the FAM and HEX channels of the QX200 reader (Bio-Rad, Montréal, QC, Canada).

### 2.7. NA Susceptibility Assays

The phenotype of resistance to OS carboxylate was evaluated by NA inhibition assays as previously described [34]. Stock virus (A/H3N2-WT used as control) and viruses isolated from mouse lungs on day 15 p.i. (previously passaged once in ST6-GalI-MDCK cells) were standardized to an NA activity level 10-fold higher than that of 2′-(4-methylumbelliferyl)-α-D-N-acetylneuraminic acid (MUNANA; Sigma, St-Louis, MO, USA) substrate (final concentration of 100 µM). The 50% inhibitory concentrations (IC_50_s) were determined from the dose–response curves. Fluorescence was measured with excitation and emission filters of 355 and 460 nm, respectively, on a Victor X3 multilabel plate reader (PerkinElmer, Waltham, MA, USA).

### 2.8. Statistical Analyses

Lung viral titers (LVTs) and levels of cellular proliferation were compared by one-way ANOVA analysis of variance, with the Dunnett’s multiple comparison post-test. A Log-Rank (Mantel–Cox) test was used to compare the Kaplan–Meier survival plots. For weight loss, the area under the curve (AUC) was calculated for each group of mice receiving the different treatments. Comparisons of AUC data and absolute cell counts among animals treated or not with CP were done using the Student’s *t*-test.

## 3. Results

### 3.1. CP-Induced Immunosuppression in Mice

Our data confirmed that CP treatment induced immunosuppressive effects in mice as demonstrated by the strong suppression of splenic T and B cell proliferative responses (Appendix A.1, Figure S1). In addition, reduced numbers of total T/B lymphocytes and neutrophils were recorded (Appendix A.2, Figure S2).

### 3.2. Efficacy of Single and Combined Therapies

**Figure 1:**
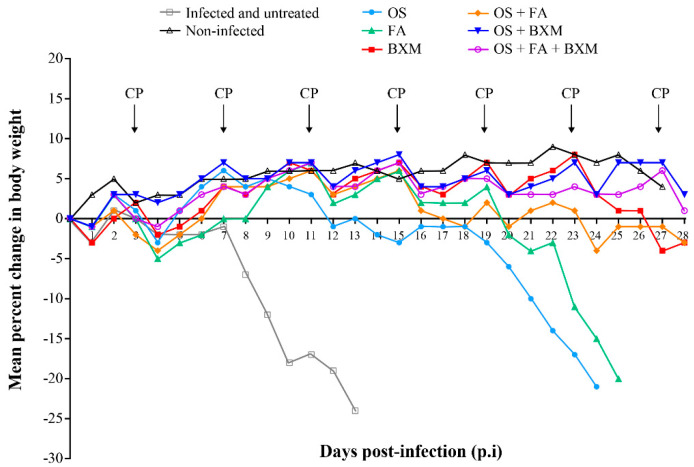
Effects of single and combined therapies on the weight of immunosuppressed (IS) mice infected with an A/H3N2 influenza virus. C57BL/6 mice intranasally (i.n) infected with 3 × 10^3^ PFU of a mouse-adapted A/Switzerland/9715293/2013 (H3N2) influenza virus received meglumine (placebo), 20 mg/kg of oseltamivir (OS; twice a day (BID)), 100 mg/kg of favipiravir (FA; BID), or 40 mg/kg of baloxavir marboxil (BXM; once a day), bi-therapy with OS and FA, OS and BXM or tri-therapy with OS, FA and BXM, starting at 48 h post-infection (p.i.) for 10 days. An uninfected group (meglumine) was added as the control. Animals were monitored daily for 28 days for weight loss and sacrificed when they lost ≥ 20% of their original body weight.

In IS mice infected with the mouse-adapted A/Switzerland/9715293/2013 (H3N2) virus, the body weight loss was observed earliest in the untreated group (on day 7 p.i.), whereas the OS- or FA-treated mice started losing body weight on days 18 and 20 p.i., respectively (Figure 1). In contrast, monotherapy with BXM significantly prevented the body weight loss compared to non-treated animals (*p* < 0.0001).

**Figure 2:**
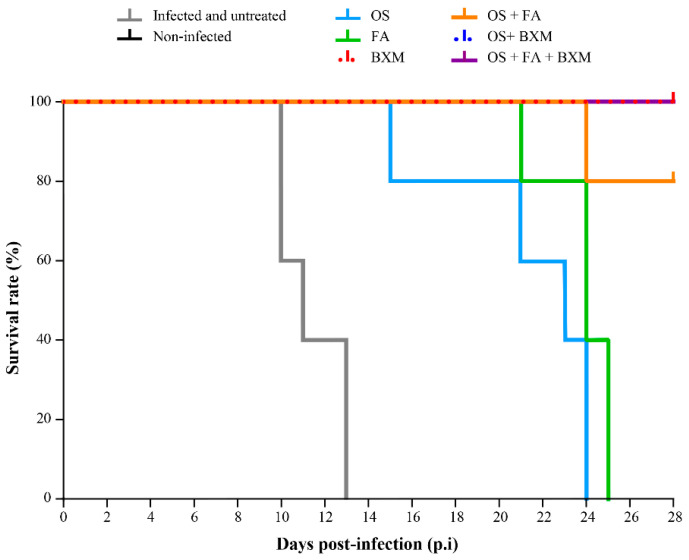
Effects of single and combined therapies on the survival of IS mice infected with an A/H3N2 influenza virus. C57BL/6 mice intranasally (i.n) infected with 3 × 10^3^ PFU of a mouse-adapted A/Switzerland/9715293/2013 (H3N2) influenza virus received meglumine (placebo), 20 mg/kg of oseltamivir (OS; twice a day (BID)), 100 mg/kg of favipiravir (FA; BID), or 40 mg/kg of baloxavir marboxil (BXM; once a day), bi-therapy with OS and FA, OS and BXM or tri-therapy with OS, FA and BXM, starting at 48 h post-infection (p.i.) for 10 days. An uninfected group (meglumine) was added as the control. Kaplan–Meier survival curves for IS mice were compared using the Log-Rank (Mantel–Cox) test.

All untreated but infected animals died between days 10 and 13 p.i. with a mean day of death (MDD) of 11.4 ± 1.5 (Figure 2). Single therapy with OS or FA did not prevent death, but significantly delayed the mortality with MDDs of 21.4 ± 3.7 (*p* = 0.0025), and 23.8 ± 1.6 (*p* = 0.0025), respectively, compared to the untreated group. BXM provided a 100% survival rate, suggesting that the efficacy of daily doses of BXM is superior to that of OS or FA (Figure 2). The combination treatment of OS and FA significantly increased the survival of mice (80%) compared to OS with an MDD of 24 days, demonstrating evident synergistic effects for this combination therapy (Figure 2). Treatment with BXM in combination with OS was not more effective than BXM alone, suggesting there was no advantage of combining these two drugs in this animal model for improving survival. The triple therapy of OS, FA, and BXM fully protected infected mice from death, resulting in a 100% survival rate (Figure 1 and Figure 2). Again, triple combination was not more effective than BXM alone.

**Figure 3:**
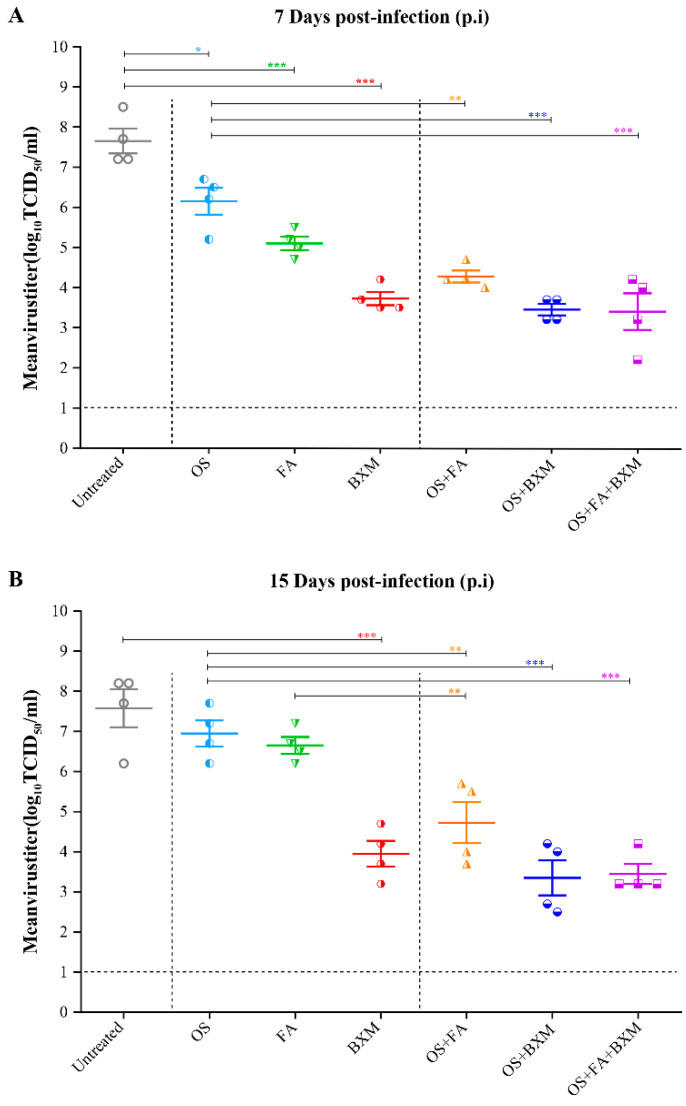
Effects of single and combined therapies on the lung viral titers of IS mice infected with an A/H3N2 influenza virus. C57BL/6 mice intranasally (i.n) infected with 3 × 10^3^ PFU of a mouse-adapted A/Switzerland/9715293/2013 (H3N2) influenza virus received meglumine (placebo), 20 mg/kg of oseltamivir (OS; twice a day (BID)), 100 mg/kg of favipiravir (FA; BID), or 40 mg/kg of baloxavir marboxil (BXM; once a day), bi-therapy with OS and FA, OS and BXM or tri-therapy with OS, FA and BXM, starting at 48 h post-infection (p.i.) for 10 days. An uninfected group (meglumine) was added as the control. Lung viral titers (LVTs) were determined by TCID50 using ST6-GalI-MDCK cells for groups of four mice euthanized on days 7 (**A**), and 15 (**B**) p.i. and compared by one-way ANOVA with Dunnett’s multiple comparisons test. * *p* < 0.05, ** *p* < 0.01, and *** *p* < 0.001.

In order to evaluate the level of virus clearance, four IS mice per group were infected and treated as described above and LVTs were evaluated on days 7 and 15 p.i. On day 7 p.i., the viral titers of IS mice treated with OS, FA, and BXM monotherapies were statistically lower than those of non-treated mice, with mean titers of 10^6.2^ (*p* < 0.05), 10^5.1^ (*p* < 0.001), and 10^3.7^ TCID_50_/mL (*p* < 0.001), respectively, compared to 10^7.6^ TCID_50_/mL for the untreated mice (Figure 3A). At this time-point, the combination therapies of OS plus FA, OS plus BXM or OS plus FA plus BXM induced a significant reduction in titers compared to the OS monotherapy with mean titers of 10^4.3^ (*p* < 0.01), 10^3.5^ (*p* < 0.001), and 10^3.4^ TCID_50_/mL (*p* < 0.001), respectively, versus 10^6.2^ TCID_50_/mL (Figure 3A).

On day 15 p.i., the LVTs remained significantly reduced in the BXM group compared to untreated animals (10^7.6^ versus 10^3.9^ TCID_50_/mL, *p* < 0.001). This was not the case for OS and FA monotherapies highlighting the superior efficacy of BXM (Figure 3B). In the combined OS and FA group, virus titers were significantly lower compared to those receiving OS or FA monotherapies (Figure 3B).

### 3.3. Emergence of Drug Resistance

In mice treated with OS, we detected an E119V substitution as a pure population by the Sanger sequencing of the NA gene from lung samples collected from two out of four mice on day 15. When assessed in NA inhibition assays, these E119V variants had mean IC_50_ values of 81.00 ± 0.01 nM and 80.39 ± 0.12 nM, respectively, equivalent to 192.9 and 191.4-fold increases compared to the A/H3N2-WT IC_50_ (Table 1). No mutations were detected by Sanger sequencing in the viral samples from mice treated with combination therapies (Table 1). No amino acid substitutions in the PB1 or PA genes were detected by Sanger sequencing in viruses from animals receiving FA or BXM, alone or in combination when compared to the parental virus. However, a ddRT-PCR assay designed for the detection of the I38T PA substitution associated with BXM resistance revealed that viruses from one mouse treated with BXM alone contained a higher proportion of I38T (13%) compared to the A/H3N2 WT (Table 1). These results suggest that antiviral combination may prevent the emergence of drug-resistant variants.

## 4. Discussion

To our knowledge, this study is the first to investigate the efficacy of OS in combination with viral polymerase inhibitors (FA and BXM) for the treatment of influenza A/H3N2 virus infection in IS mice. Antiviral treatments are widely used in high-risk patients to inhibit IV replication [35,36]. However, existing therapeutic regimens are based on clinical trials conducted on healthy individuals [37,38]. Pre-clinical studies using IS animals can provide additional information for optimizing antiviral protocols for high-risk patients. Here, we used a pharmacologically IS-mouse model by treating animals with CP to evaluate the efficacy of a NAI (Oseltamivir) and polymerase inhibitors (FA and BXM) combinations against influenza A/H3N2 virus infection. Treatment with CP has been reported to induce a reduction in NK cell activity, the inhibition of T and B cell proliferative responses, lessening the number of T-helper and T-suppressor cells, and decreasing cytokine and interferon production [26,27,28]. CP is well established drug is and routinely used to induce an immunosuppressive state in animals [39,40,41,42]. In addition, this mouse model is inexpensive and easy to maintain.

It has been reported that prolonged virus shedding and the emergence of NAI resistance are two phenomena commonly observed in IS patients during single antiviral treatment [13,15,16,43,44]. Therefore, combination therapy is an attractive clinical approach aimed at improving the clinical outcome and preventing the risk of drug resistance [45]. In our regimen, dosing at an OS at 20 mg/kg/day was chosen as the oral bioavailability is reportedly similar to the recommended critically ill human oral dose of 150 mg BID daily [46,47,48,49]. For FA, we selected the treatment regimens on the basis of our previous in vivo protection study with A/H1N1pdm virus in IS mice (24). Our data demonstrate that despite the significant delay in mortality induced by OS or FA monotherapies, all mice that received such treatments eventually died with high LVTs suggesting that these drugs given as monotherapy failed to achieve virus clearance and prevent death.

In comparison with either monotherapy, we observed a synergistic interaction between OS and FA combination therapy; indeed, 80% of mice that received this treatment survived. OS/FA-treated mice displayed a significantly lower body weight loss and viral load compared to those of the monotherapies. These results are in line with previous experiments by our group [24] and others [25] on seasonal A/H1N1pdm viruses. We previously reported that the combination therapy of OS (20 mg/kg) and FA (50 mg/kg) for 10 days in IS mice infected with recombinant A/Québec/144147/09 (H1N1pdm) virus significantly delayed mortality and reduced the LVTs compared to the treatment with a single drug regimen [24]. In addition, Kiso et al. [25] also showed that the combination therapy of OS (25 mg/kg) and FA (20 or 30 mg/kg) for 28 days started one hour p.i. increased survival time in nude mice infected with mouse-adapted A/California/04/2009 (H1N1pdm) virus; however, this combination failed to achieve virus clearance, resulting in mortality after the termination of treatment.

The highest concentration of FA applied in our regimens to treat IS mice infected with A/H3N2 virus (100 mg/kg) versus A/H1N1pdm viruses (30 or 50 mg/kg) in the previous studies might explain the synergistic effect observed in our study, suggesting that ≥100 mg/kg of FA is required to increase the efficacy of such therapy. Overall, our results suggest the use of FA in combination with a NAI, as a rational choice for the treatment of severe seasonal influenza A/H1N1 and A/H3N2 virus infections.

It has been suggested that oral administration of 15 mg/kg of BXM BID for 5 days in immunocompetent mice can mimic the target plasma concentration (6.85 ng/mL) of BXA in humans [7,50,51]. Therefore, the efficacy of treatment with BXM alone or in combination was evaluated at 40 mg/kg once daily for 10 days in our IS mouse model to predict clinical effectiveness. In addition, Hirotsu and collaborators [52] also suggest that repeated BXM dosing may reduce the risk of variant virus emergence.

Interestingly, we found that 40 mg/kg of BXM once a day for 10 days provided the full protection of IS mice, significantly reduced the body weight loss and the viral load in comparison with the untreated group, indicating that BXM might provide superior protection than OS or FA monotherapies at the doses used in this experiment. In a recent study in IS mice treated with CP, Fukao et al. [53] showed a strong efficacy of BXM treatment (15 or 50 mg/kg; BID for 5 days) against A/PR/8/34 (H1N1) virus, with a significant reduction in LVTs and weight loss when the treatment was delayed by up to 120 h p.i. In contrast, a recent study in nude mice has shown that 28 days of BXM monotherapy (10 mg/kg once daily) against A(H1N1)pdm09 virus prolonged the median survival time to 49 days, in comparison to 5 days in the untreated controls, but did not decrease LVTs with all mice dying after the completion of treatment [54].

The difference in protection efficacy between these studies probably lies in the extent of immune suppression between the animals. Indeed, unlike nude mice, CP-administration leads to a less drastic immune suppression, which potentially more closely mimics immunosuppression in most individuals. Moreover, there was a difference between BXM doses and subtypes (10 mg/kg; A(H1N1)pdm09 versus 40 mg/kg; A/H3N2). In the present study, no difference in terms of weight loss, survival, and LVTs were shown when BXM was used as monotherapy or combined with OS and/or FA. This could be explained by the higher dose of BXM (40 mg/kg) used herein. Further work using different BXM regimens and other animal models, such as IS ferrets is needed.

The emergence of drug-resistant variants is a major concern, particularly in high-risk populations. In the current study, OS and BXM monotherapies induced the NA/E119V and PA/I38T substitutions that are associated with OS and BXM resistance, respectively [21,33,55]. These mutations were not found in mice that received combination treatments, suggesting that such an approach could successfully suppress the emergence of antiviral resistance in our IS animal model infected with the A/H3N2 virus. In contrast, the previous study by Kiso et al. [25] demonstrated that the combination therapy did not prevent the emergence of NA inhibitor-resistant variants, which can be explained again by different levels of immunosuppression and drug concentrations. Compared with our previous study [24], we observed a significant decrease in LVTs with combination therapies, which could explain the absence of drug-resistant variants in IS mice. We found no viruses with mutations in the PB1 gene from mice treated with FA alone or in combination, supporting a high genetic barrier to resistance as previously observed [24,25]. More surprising is the absence of resistance seen with BXM combinations, which may be related to our drug regimen. However, we did not perform a deep sequencing of the polymerase genes, which is a limitation of our study.

## 5. Conclusions

In summary, our results highlighted the efficacy of combination therapy with OS and polymerase inhibitors (FA and BXM) to reduce the incidence of resistance during A/H3N2 infection in an IS mouse model. These drug regimens should be validated in other animal models such as ferrets and guinea pigs and eventually in clinical trials.

## Figures and Tables

**Table 1:** Phenotypic and genotypic analyses of drug resistance in lung viral samples of IS mice.

	NAI Assay				% Mut PA/I38T by dd-PCR ^d^
Sample No. ^a^	Treatment ^b^	Mean IC_50_ ± SD (nM)	Fold	NA Mutations	
**-**	**^c^ A/H3N2-WT**	0.42 ± 0.05	-	-	0
**1**	**Non-treated**	0.47 ± 0.07	1.1	-	NA ^e^
**2**		0.43 ± 0.11	1.0	-	
**3**		0.9 ± 0.03	2.1	-	
**4**		0.43 ± 0.10	1.0	-	
**1**	**OS**	81 ± 0.01	192.9	**E119V**	NA
**2**		4.43 ± 0.55	10.6	-	
**3**		4.3 ± 1.56	10.2	-	
**4**		80.39 ± 0.12	191.4	**E119V**	
**1**	**OS + FA**	2.78 ± 0.18	6.6	-	NA
**2**		1.13 ± 0.11	2.7	-	
**3**		3.64 ± 1.46	8.7	-	
**4**		4.37 ± 0.65	10.4	-	
**1**	**OS + BXM**	3.51 ± 0.51	8.3	-	0
**2**		1.61 ± 0.54	3.8	-	0
**3**		0.95 ± 0.01	2.3	-	0
**4**		2.56 ± 1.41	6.1	-	0
**1**	**OS + FA + BXM**	3.3 ± 0.27	7.9	-	0
**2**		1.03 ± 0.09	2.5	-	0
**3**		1.01 ± 0.91	2.4	-	0
**4**		0.68 ± 0.02	1.6	-	0
**1**	**BXM**	NA			**13**
**2**					0
**3**					0
**4**					0

^a^ samples were collected from the individual lungs of IS mice infected with A/H3N2 virus on day 15 post-infection. ^b^ Treatment: OS (pseltamivir; 20 mg/kg/BID); FA (favipiravir; 100 mg/kg/BID); BXM (baloxavir marboxil; 40 mg/kg/once a day). ^c^ A/H3N2-WT: A/Switzerland/9715293/2013 (H3N2). ^d^ mutation (PA/I38T) frequency obtained from Droplet digital PCR (ddRT-PCR) of the groups of mice treated with BXM monotherapy or in combination with OS or FA. ^e^ NA: not applicable. NAI; neuraminidase inhibition assay, IC_50_; 50% inhibitory concentration, Mut; mutation.

## Supplementary Materials

The following are available online at https://www.mdpi.com/2076-2607/8/12/1968/s1, Figure S1. Cyclophosphamide (CP) treatment impairs the proliferative response of T and B cells on day 4 p.i. Figure S2. Cyclophosphamide (CP) therapy reduces the numbers of immune splenic cells. Table S1. Antibodies used in flow cytometry analysis.

## Appendix A

### Appendix A.1

Overall, the T, and B cells proliferative capacities were strongly decreased in CP-treated mice. Following CP therapy, the percentage of proliferating T cells significantly decreased on day 4 p.i. from 92.2% to 63.1% in uninfected mice (*p* < 0.001) and from 93.8% to 72.6% in infected mice (*p* < 0.01). CP therapy also led to an inhibition of B cells proliferative capacity. Indeed, CP injection significantly reduced the frequency of proliferating B cells on day 4 post infection from 82.5% to 36.2% in uninfected mice (*p* < 0.05), and from 94.7% to 40.7% in infected animals (*p* < 0.01). In contrast, no significant decline in NK proliferative capacity was observed following CP administration. Similar decreases in T and B cells proliferation were also noted after CP therapy on day 10 p.i. while no effect on NK proliferative function was observed on day 10 p.i.

### Appendix A.2

Treatment with CP significantly reduced the numbers of T, B, and neutrophils cells in uninfected animals on day 4 and 10 p.i. In infected mice receiving CP, splenic T cells frequency was only significantly reduced on day 10 p.i. All CP-treated animals infected or not with A/H3N2 virus had significantly reduced numbers of B cells on day 4 p.i. while on 10-day p.i, this depletion only reached statistical significance in uninfected mice. Treatment with CP significantly reduced the number of neutrophils in uninfected animals on days 4 and 10 p.i. However, in infected mice receiving CP, neutrophils were significantly reduced only on day 10 p.i. Although, there was a trend toward lower NK, dendritic, and monocyte cells in both infected and non-infected mice, such decrease was not always statistically significant.

## References

[1] Simonsen L., Clarke M.J., Williamson G.D., Stroup D.F., Arden N.H., Schonberger L.B. (1997). The impact of influenza epidemics on mortality: Introducing a severity index. Am. J. Public Health.

[2] Beard K.R., Brendish N.J., Clark T.W. (2018). Treatment of influenza with neuraminidase inhibitors. Curr. Opin. Infect. Dis..

[3] Schirmer P., Holodniy M. (2009). Oseltamivir for treatment and prophylaxis of influenza infection. Expert Opin. Drug Saf..

[4] Lee L.Y.Y., Zhou J., Frise R., Goldhill D.H., Koszalka P., Mifsud E.J., Baba K., Noda T., Ando Y., Sato K. (2020). Baloxavir treatment of ferrets infected with influenza A(H1N1)pdm09 virus reduces onward transmission. PLoS Pathog..

[5] Hayden F.G., Shindo N. (2019). Influenza virus polymerase inhibitors in clinical development. Curr. Opin. Infect. Dis..

[6] Noshi T., Kitano M., Taniguchi K., Yamamoto A., Omoto S., Baba K., Hashimoto T., Ishida K., Kushima Y., Hattori K. (2018). In vitro characterization of baloxavir acid, a first-in-class cap-dependent endonuclease inhibitor of the influenza virus polymerase PA subunit. Antivir. Res..

[7] Hayden F.G., Sugaya N., Hirotsu N., Lee N., de Jong M.D., Hurt A.C., Ishida T., Sekino H., Yamada K., Portsmouth S. (2018). Baloxavir Marboxil for Uncomplicated Influenza in Adults and Adolescents. N. Engl. J. Med..

[8] Ison M.G., Portsmouth S., Yoshida Y., Shishido T., Mitchener M., Tsuchiya K., Uehara T., Hayden F.G. (2020). Early treatment with baloxavir marboxil in high-risk adolescent and adult outpatients with uncomplicated influenza (CAPSTONE-2): A randomised, placebo-controlled, phase 3 trial. Lancet Infect Dis.

[9] Zaraket H., Saito R. (2016). Japanese Surveillance Systems and Treatment for Influenza. Curr. Treat Options Infect. Dis..

[10] Sangawa H., Komeno T., Nishikawa H., Yoshida A., Takahashi K., Nomura N., Furuta Y. (2013). Mechanism of action of T-705 ribosyl triphosphate against influenza virus RNA polymerase. Antimicrob. Agents Chemother..

[11] Goldhill D.H., Te Velthuis A.J.W., Fletcher R.A., Langat P., Zambon M., Lackenby A., Barclay W.S. (2018). The mechanism of resistance to favipiravir in influenza. Proc. Natl. Acad. Sci. USA.

[12] Memoli M.J., Athota R., Reed S., Czajkowski L., Bristol T., Proudfoot K., Hagey R., Voell J., Fiorentino C., Ademposi A. (2014). The natural history of influenza infection in the severely immunocompromised vs nonimmunocompromised hosts. Clin. Infect. Dis..

[13] Baz M., Abed Y., McDonald J., Boivin G. (2006). Characterization of multidrug-resistant influenza A/H3N2 viruses shed during 1 year by an immunocompromised child. Clin. Infect. Dis..

[14] Memoli M.J., Hrabal R.J., Hassantoufighi A., Jagger B.W., Sheng Z.M., Eichelberger M.C., Taubenberger J.K. (2010). Rapid selection of a transmissible multidrug-resistant influenza A/H3N2 virus in an immunocompromised host. J. Infect. Dis..

[15] Roussy J.F., Abed Y., Bouhy X., Boivin G. (2013). Emergence of an oseltamivir-resistant influenza A/H3N2 virus in an elderly patient receiving a suboptimal dose of antiviral prophylaxis. J. Clin. Microbiol..

[16] Renaud C., Boudreault A.A., Kuypers J., Lofy K.H., Corey L., Boeckh M.J., Englund J.A. (2011). H275Y mutant pandemic (H1N1) 2009 virus in immunocompromised patients. Emerg. Infect. Dis..

[17] Tamura D., DeBiasi R.L., Okomo-Adhiambo M., Mishin V.P., Campbell A.P., Loechelt B., Wiedermann B.L., Fry A.M., Gubareva L.V. (2015). Emergence of Multidrug-Resistant Influenza A(H1N1)pdm09 Virus Variants in an Immunocompromised Child Treated With Oseltamivir and Zanamivir. J. Infect. Dis..

[18] Baz M., Abed Y., Papenburg J., Bouhy X., Hamelin M.E., Boivin G. (2009). Emergence of oseltamivir-resistant pandemic H1N1 virus during prophylaxis. N. Engl. J. Med..

[19] Mitha E., Krivan G., Jacobs F., Nagler A., Alrabaa S., Mykietiuk A., Kenwright A., Le Pogam S., Clinch B., Vareikiene L. (2019). Safety, Resistance, and Efficacy Results from a Phase IIIb Study of Conventional- and Double-Dose Oseltamivir Regimens for Treatment of Influenza in Immunocompromised Patients. Infect. Dis. Ther..

[20] Tamura D., Sugaya N., Ozawa M., Takano R., Ichikawa M., Yamazaki M., Kawakami C., Shimizu H., Uehara R., Kiso M. (2011). Frequency of drug-resistant viruses and virus shedding in pediatric influenza patients treated with neuraminidase inhibitors. Clin. Infect. Dis..

[21] Okomo-Adhiambo M., Demmler-Harrison G.J., Deyde V.M., Sheu T.G., Xu X., Klimov A.I., Gubareva L.V. (2010). Detection of E119V and E119I mutations in influenza A (H3N2) viruses isolated from an immunocompromised patient: Challenges in diagnosis of oseltamivir resistance. Antimicrob. Agents Chemother..

[22] Meijer W.J., Kromdijk W., van den Broek M.P., Haas P.J., Minnema M.C., Boucher C.A., de Lange D.W., Wensing A.M. (2015). Treatment of Immunocompromised, Critically Ill Patients with Influenza A H1N1 Infection with a Combination of Oseltamivir, Amantadine, and Zanamivir. Case Rep. Infect. Dis..

[23] Dunning J., Baillie J.K., Cao B., Hayden F.G. (2014). Antiviral combinations for severe influenza. Lancet Infect. Dis..

[24] Baz M., Carbonneau J., Rheaume C., Cavanagh M.H., Boivin G. (2018). Combination Therapy with Oseltamivir and Favipiravir Delays Mortality but Does Not Prevent Oseltamivir Resistance in Immunodeficient Mice Infected with Pandemic A(H1N1) Influenza Virus. Viruses.

[25] Kiso M., Lopes T.J.S., Yamayoshi S., Ito M., Yamashita M., Nakajima N., Hasegawa H., Neumann G., Kawaoka Y. (2018). Combination Therapy With Neuraminidase and Polymerase Inhibitors in Nude Mice Infected With Influenza Virus. J. Infect. Dis..

[26] Mastino A., Grelli S., Premrov M.G., Favalli C. (1991). Susceptibility to influenza A virus infection in mice immunosuppressed with cyclophosphamide. J. Chemother..

[27] Singer S.H., Noguchi P., Kirschstein R.L. (1972). Respiratory diseases in cyclophosphamide-treated mice. II. Decreased virulence of PR8 influenza virus. Infect. Immun..

[28] Hurd J., Heath R.B. (1975). Effect of cyclophosphamide on infections in mice caused by virulent and avirulent strains of influenza virus. Infect. Immun..

[29] Baz M., M’Hamdi Z., Carbonneau J., Lavigne S., Couture C., Abed Y., Boivin G. (2019). Synergistic PA and HA mutations confer mouse adaptation of a contemporary A/H3N2 influenza virus. Sci. Rep..

[30] Hatakeyama S., Sakai-Tagawa Y., Kiso M., Goto H., Kawakami C., Mitamura K., Sugaya N., Suzuki Y., Kawaoka Y. (2005). Enhanced expression of an alpha2,6-linked sialic acid on MDCK cells improves isolation of human influenza viruses and evaluation of their sensitivity to a neuraminidase inhibitor. J. Clin. Microbiol..

[31] Reed L.M.H. (1938). A simple method of estimating fifty percent endpoints. Am. J. Hyg.

[32] Taylor S.C., Carbonneau J., Shelton D.N., Boivin G. (2015). Optimization of Droplet Digital PCR from RNA and DNA extracts with direct comparison to RT-qPCR: Clinical implications for quantification of Oseltamivir-resistant subpopulations. J. Virol. Methods.

[33] Checkmahomed L., M’Hamdi Z., Carbonneau J., Venable M.C., Baz M., Abed Y., Boivin G. (2020). Impact of the Baloxavir-Resistant Polymerase Acid I38T Substitution on the Fitness of Contemporary Influenza A(H1N1)pdm09 and A(H3N2) Strains. J. Infect. Dis..

[34] Baz M., Abed Y., Boivin G. (2007). Characterization of drug-resistant recombinant influenza A/H1N1 viruses selected in vitro with peramivir and zanamivir. Antivir. Res..

[35] Muthuri S.G., Venkatesan S., Myles P.R., Leonardi-Bee J., Al Khuwaitir T.S., Al Mamun A., Anovadiya A.P., Azziz-Baumgartner E., Baez C., Bassetti M. (2014). Effectiveness of neuraminidase inhibitors in reducing mortality in patients admitted to hospital with influenza A H1N1pdm09 virus infection: A meta-analysis of individual participant data. Lancet Respir. Med..

[36] Oboho I.K., Reed C., Gargiullo P., Leon M., Aragon D., Meek J., Anderson E.J., Ryan P., Lynfield R., Morin C. (2016). Benefit of Early Initiation of Influenza Antiviral Treatment to Pregnant Women Hospitalized With Laboratory-Confirmed Influenza. J. Infect. Dis..

[37] Thorlund K., Awad T., Boivin G., Thabane L. (2011). Systematic review of influenza resistance to the neuraminidase inhibitors. BMC Infect Dis..

[38] Dobson J., Whitley R.J., Pocock S., Monto A.S. (2015). Oseltamivir treatment for influenza in adults: A meta-analysis of randomised controlled trials. Lancet.

[39] Steel G.G., Peckham M.J. (1980). Human tumour xenografts: A critical appraisal. Br. J. Cancer Suppl..

[40] Smee D.F., Dagley A., Downs B., Hagloch J., Tarbet E.B. (2015). Enhanced efficacy of cidofovir combined with vaccinia immune globulin in treating progressive cutaneous vaccinia virus infections in immunosuppressed hairless mice. Antimicrob. Agents Chemother..

[41] Floersheim G.L., Bieri A., Chiodetti N. (1986). Xenografts in pharmacologically immunosuppressed mice as a model to test the chemotherapeutic sensitivity of human tumors. Int. J. Cancer.

[42] Goodman M.M., McCullough J.L., Biren C.A., Barr R.J. (1987). A model of human melanoma in cyclosporine-immunosuppressed rats. J. Investig. Dermatol..

[43] Hurt A.C., Leang S.K., Tiedemann K., Butler J., Mechinaud F., Kelso A., Downie P., Barr I.G. (2013). Progressive emergence of an oseltamivir-resistant A(H3N2) virus over two courses of oseltamivir treatment in an immunocompromised paediatric patient. Influenza Other Respir. Viruses.

[44] Memoli M.J., Hrabal R.J., Hassantoufighi A., Eichelberger M.C., Taubenberger J.K. (2010). Rapid selection of oseltamivir- and peramivir-resistant pandemic H1N1 virus during therapy in 2 immunocompromised hosts. Clin. Infect. Dis..

[45] Tsiodras S., Mooney J.D., Hatzakis A. (2007). Role of combination antiviral therapy in pandemic influenza and stockpiling implications. BMJ.

[46] Sharma G., Champalal Sharma D., Hwei Fen L., Pathak M., Bethur N., Pendharkar V., Peiris M., Altmeyer R. (2013). Reduction of influenza virus-induced lung inflammation and mortality in animals treated with a phosophodisestrase-4 inhibitor and a selective serotonin reuptake inhibitor. Emerg. Microbes Infect..

[47] Flannery A.H., Thompson Bastin M.L. (2014). Oseltamivir Dosing in Critically Ill Patients With Severe Influenza. Ann. Pharmacother..

[48] Ward P., Small I., Smith J., Suter P., Dutkowski R. (2005). Oseltamivir (Tamiflu) and its potential for use in the event of an influenza pandemic. J. Antimicrob. Chemother..

[49] South East Asia Infectious Disease Clinical Research Network (2013). Effect of double dose oseltamivir on clinical and virological outcomes in children and adults admitted to hospital with severe influenza: Double blind randomised controlled trial. BMJ.

[50] Fukao K., Noshi T., Yamamoto A., Kitano M., Ando Y., Noda T., Baba K., Matsumoto K., Higuchi N., Ikeda M. (2019). Combination treatment with the cap-dependent endonuclease inhibitor baloxavir marboxil and a neuraminidase inhibitor in a mouse model of influenza A virus infection. J. Antimicrob. Chemother..

[51] Koshimichi H., Ishibashi T., Kawaguchi N., Sato C., Kawasaki A., Wajima T. (2018). Safety, Tolerability, and Pharmacokinetics of the Novel Anti-influenza Agent Baloxavir Marboxil in Healthy Adults: Phase I Study Findings. Clin. Drug Investig..

[52] Hirotsu N., Sakaguchi H., Sato C., Ishibashi T., Baba K., Omoto S., Shishido T., Tsuchiya K., Hayden F.G., Uehara T. (2020). Baloxavir Marboxil in Japanese Pediatric Patients With Influenza: Safety and Clinical and Virologic Outcomes. Clin. Infect. Dis..

[53] Fukao K., Ando Y., Noshi T., Kitano M., Noda T., Kawai M., Yoshida R., Sato A., Shishido T., Naito A. (2019). Baloxavir marboxil, a novel cap-dependent endonuclease inhibitor potently suppresses influenza virus replication and represents therapeutic effects in both immunocompetent and immunocompromised mouse models. PLoS ONE.

[54] Kiso M., Yamayoshi S., Murakami J., Kawaoka Y. (2019). Baloxavir marboxil treatment of nude mice infected with influenza A virus. J. Infect. Dis..

[55] Eshaghi A., Shalhoub S., Rosenfeld P., Li A., Higgins R.R., Stogios P.J., Savchenko A., Bastien N., Li Y., Rotstein C. (2014). Multiple influenza A (H3N2) mutations conferring resistance to neuraminidase inhibitors in a bone marrow transplant recipient. Antimicrob. Agents Chemother..

