# Supplementary material for: Effects of Different Drug Combinations in Immunodeficient Mice Infected with an Influenza A/H3N2 Virus
Source: Microorganisms. 2020 Dec 11;8(12):1968. doi: 10.3390/microorganisms8121968 (PMC7764069; doi:10.3390/microorganisms8121968)
Supplement: Supplementary file 1 [file microorganisms-08-01968-s001.pdf]

Supplementary Materials:

Table S1. Antibodies used in flow cytometry analysis.

	**Target**	**Clone**	**Fluorochrome**	**Manufacturer**
**Flow cytometry Quantification**	CD45	30-F11	APC-Cy7	BD Biosciences
	CD11b	M1/70	BUV737	BD Biosciences
	CD11c	HL3	Pe-Cy7	BD Biosciences
	NK 1.1	PK136	BUV395	BD Biosciences
	NKp46	NCR1	PE	eBioscience
	B220	RA3-6B2	PerCP-Cy5.5	BD Biosciences
	Ly6C	AL-21	BV605	BD Biosciences
	Ly6G	1A8	FITC	BD Biosciences
	CD4	GK1.5	BV421	BD Biosciences
	CD8a	53-6.7	BUV395	eBioscience
	CD3ε	145-2C11	PE-CF594	BD Biosciences

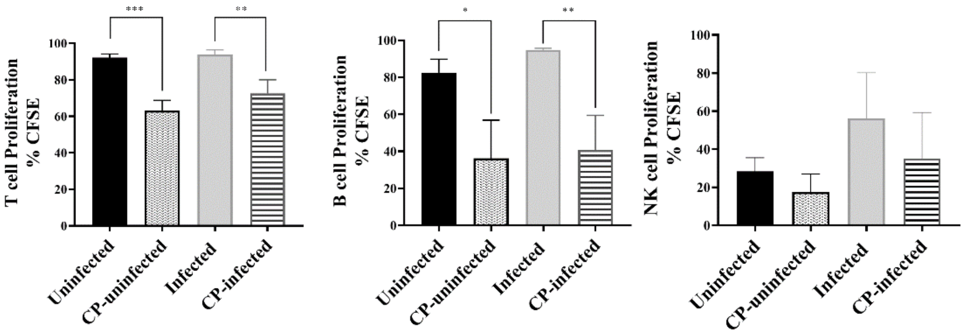

Figure S1. Cyclophosphamide (CP) treatment impairs the proliferative response of T and B cells on day 4 p.i. Single-cell suspensions were obtained from mouse splenocytes by homogenization using a syringe and a cell strainer. Cells were stained with 5 μM CFSE (carboxyfluorescein succinimidyl ester) according to the manufacturer's instructions (Thermo Fisher Scientific, Burlington, VT, USA). For T cells activation, splenocytes were stimulated with plate-bound anti-CD3ε (145-2C11; 1 μg/mL) and CD28 (37.51; 1 μg/mL) monoclonal antibodies (BD Biosciences). To assess B and NK cells activation, splenocytes were stimulated with 1 μg/mL of resiquimod (R848), and 100 ng/mL interleukin IL-15, respectively (Sigma Aldrich, ON, Canada). To study proliferation five days post-stimulation, splenocytes were washed and incubated with BD Horizon™ Fixable Viability Stain 510 (FVS510; BD Biosciences) for dead cell exclusion, and stained with anti-CD3ε (145-2C11), B220 (RA3-6B2, BD Biosciences) or NK1.1 antibodies (PK136, BD Biosciences), all PERP-Cy55 conjugated. Cell proliferation was analyzed by flow cytometry (LSR-II, BD Biosciences). The frequencies of proliferating cells (CFSE Low) between groups were compared using a one-way ANOVA with Dunnett's multiple comparisons test. * $p < 0.05$, ** $p < 0.01$, and *** $p < 0.001$.

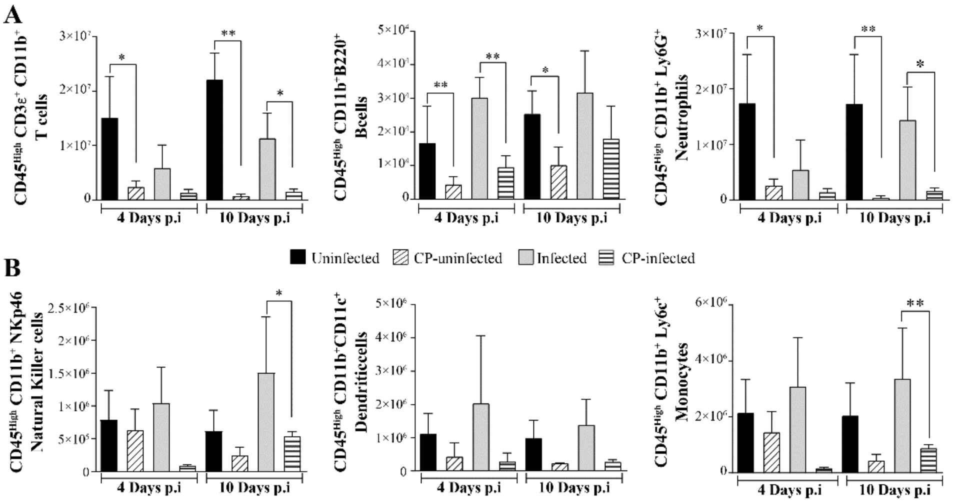

Figure S2. Cyclophosphamide (CP) therapy reduces the numbers of immune splenic cells. Spleens removed on days 4 and 10 p.i were homogenized and incubated in DPBS containing Liberase™ TL, Nα-Tosyl-L-lysine chloromethyl ketone hydrochloride (TLCK) (Sigma-Aldrich) and DNase-I (Biorbyt). To label the various immune subsets, splenocyte cell suspensions were washed and incubated with BD Horizon™ Fixable Viability Stain 510 (FVS510; BD Biosciences), followed by a blocking step with anti-mouse CD16/CD32 (2.4G2; BD Biosciences) to improve staining specificity. Cells were incubated with a cocktail of antibodies (Supplementary Table S1) to identify the different immune cell subsets. Labelled cells were fixed, washed and resuspended in DPBS. Finally, a volume of 50 μL of Precision Count Beads™ (BioLegend) was added to each sample in order to obtain an absolute cell count number. Flow cytometry data was analyzed by BD SORP LSR II (BD Biosciences) software. Frequencies of absolute counts for the different groups were compared using the Student's *t*-test. * $p < 0.05$, ** $p < 0.01$, and *** $p < 0.001$.
